# A SYSTEMATIC REVIEW OF NONMEDICAL FACTORS INFLUENCING CHINESE PATIENTS’ QUALITY OF LIFE IN THEIR LAST YEAR

**DOI:** 10.1093/geroni/igad104.0727

**Published:** 2023-12-21

## Abstract

Many studies have demonstrated that healthcare providers’ attention to non-medical issues and involvement in non-medical services can greatly improve the Quality of life (QOL) of dying individuals. Over the past decades, China has made great progress in providing the best possible care for the dying. However, the experiences of Chinese dying persons and their families in the last year of life have not been adequately synthesized. Thus, this systematic review aimed to summarize the last-year experiences of the Chinese dying persons and their families and to identify non-medical factors affecting their QOL. This review followed the PRISMA guideline. Peer-reviewed articles published before November 2022 were searched in both Chinese and English electronic databases. Studies were included if their participants had a life expectancy of one year or less, or family members acting as proxies were asked to recall patients’ experiences during their last year of life. The final review included 24 studies. The findings were classified into four aspects: (1) culture, including filial piety and the importance of family harmony; (2) emotional distress, including feeling like a burden to others, and depressive symptoms; (3) family involvement, including family support and involvement in decision-making; (4) connections to available services, including home-visit nurses and training on caregiving techniques. These findings implied that culturally appropriate non-medical interventions targeted at promoting the mental health of dying individuals, open communication in the family, and connections to available healthcare resources can greatly improve the QOL of dying Chinese persons during their final year of life.

